# Accuracy of smartwatches for the remote assessment of exercise capacity

**DOI:** 10.1038/s41598-024-74140-x

**Published:** 2024-10-03

**Authors:** Alexandra Jamieson, Siana Jones, Nishi Chaturvedi, Alun D. Hughes, Michele Orini

**Affiliations:** 1 MRC Unit for Lifelong Health and Ageing, UCL, 5th Floor, 1-19 Torrington Place, London, WC1E 7HB UK; 2 Department of Biomedical Engineering, King’s College London, London, UK

**Keywords:** Population screening, Data acquisition

## Abstract

Exercise capacity is a strong independent predictor of cardiovascular and all-cause mortality. The utilization of well-established submaximal tests of exercise capacity such as the 6-min walk test (6MWT), 3-min step test (3MST) and 10-chair rise test (10CRT) in the community would improve patient care but requires remote monitoring technology. Consumer grade smartwatches provide such an opportunity, however, their accuracy in measuring physiological responses to these tests is unclear. The aim of this study was to determine the accuracy of consumer grade smartwatches in assessing exercise capacity to develop a framework for remote, unsupervised testing. 16 healthy adults (7 male (44%), age median 27 [interquartile range (IQR) 26,29] years) performed 6MWTs using two protocols: (1) standard—straight 30 m laps (6MWT-standard) and 2) continuous lap—circular 240 m laps around a park (6MWT-continuous lap), 3MSTs and 10CRTs. Each one of these four tests was performed three times across two clinic visits. Each participant was fitted with a Garmin Vivoactive4 and Fitbit Sense smartwatch to measure three parameters: distance, step counts and heart rate (HR) response. Reference measures were a meter-wheel, hand tally counter and ECG, respectively. Mean HR was measured at rest, peak exercise and recovery. Agreement was measured using Bland–Altman analysis for repeated measures and summarized as median absolute percentage errors (MAPE). Distance during 6MWT-continuous lap had better agreement than during 6MWT-standard for both Garmin (MAPE: 6.4% [3.0, 10.4%] versus 20.1% [13.9, 28.4%], *p* < 0.001) and Fitbit (8.0% [2.9, 10.1% versus 18.8% [15.2, 28.1%], *p* < 0.001). Garmin measured step count more accurately than Fitbit (MAPE: 1.8% [0.9, 2.9%] versus 8.0% [2.6, 12.3%], *p* < 0.001). Irrespective of test, both devices showed excellent accuracy in measuring HR at rest and recovery (≤ 3%), while accuracy decreased during peak exercise (Fitbit: ~ 12% and Garmin: ~ 7%). In young adults without mobility difficulties, exercise capacity can be measured remotely using standardized tests and consumer grade smartwatches.

## Introduction

Exercise capacity, defined as the maximal or sub-maximal amount of physical exertion that an individual can sustain during a designated exercise test, is a strong independent predictor of cardiovascular and all-cause mortality and is a useful diagnostic and prognostic health indicator for patients in clinical and research settings alike^1^.

Well-established standardized tests include the 6-min walk test (6MWT), 3-min step test (3MST) and the 10-chair rise test (10CRT)^2–4^.The 6MWT is a useful, simple and easy to administer sub-maximal test that correlates with well-established indices of cardiorespiratory fitness (CRF) and is widely used to assess the responses to treatment interventions in patients with cardiovascular and pulmonary diseases^2^. Furthermore, the distance covered during a self-paced 6-min walk is an independent prognostic indicator^5,6^. The unsupervised use of this test in the community would be beneficial, however, a modified protocol allowing individuals to walk freely instead of along 30 m straight paths is required.

The Tecumseh 3MST combined with measurement of heart rate can be utilized to estimate indices of CRF with acceptable accuracy^7–9^ and precision^10,11^. Heart or pulse rate recovery measurements from the first 30–60 s post-exercise, have been shown to correlate with risk factors^3,12^.

The 10-chair rise test (10CRT) is a well-established functional assessment of exercise capacity which requires lower body strength and power in addition to balance and coordination and is routinely used in comprehensive geriatric assessments and research fields alike.

These validated tests are usually conducted in a clinical setting supervised by trained personnel and require significant time investment. These constraints restrict the frequency at which they can be performed and their use for risk prediction at the population level and in very large epidemiological studies.

Novel wrist-worn wearable technologies use tri-axial accelerometers, photoplethysmography (PPG) sensors and proprietary algorithms to measure movement behaviours and associated physiological changes such as heart rate, distance, and step count. In the context of healthcare, these devices provide an opportunity to monitor such parameters outside of the clinical environment, at more frequent or regular intervals, at scale. This would enable the identification of trends over time, without increased staff/patient burden and associated costs^13–15^. Yet, there are very few studies validating the use of such technology to perform standardized tests of exercise capacity in the community setting using smartwatches.

A recent study used the Apple smartwatch and iPhone to compare data collected during supervised 6MWTs performed in clinic and 6MWTs performed at home to assess “frailty” (defined as walking < 300 m on an in-clinic 6MWT)^16^. The study reported the home-based 6MWT to be 83% sensitive and 60% specific in assessing “frailty” with stronger associations reported using step count rather than GPS distance, but the study did not assess agreement between the smartwatch and a reference measurement outside of the clinic^16^.

The utilization of state-of-the-art consumer grade smartwatches in conjunction with well-established clinic-based tests provides an exciting opportunity for remote monitoring of exercise capacity at an unprecedented scale. However, the accuracy of such devices in this context is yet to be fully addressed. Therefore, the aim of this study was to assess the accuracy of consumer-grade smartwatches in the remote assessment of exercise capacity and heart rate (HR) response to exercise, across a range of tests. This pilot of procedures in a young and healthy population could be used to develop a framework for community-based, unsupervised, testing. A preliminary version of this work has been reported^17^.

## Methods

### Study participants

16 healthy adults (male 7(44%), age 27[26,29] years) were recruited from staff and students at University College London (UCL). Indoor and outdoor research procedures took place at the Bloomsbury Centre for Clinical Phenotyping (BCCP), UCL and Tavistock Square Gardens, London, respectively. The study was performed in accordance with the principles of the declaration of Helsinki and approved by the UCL Research Ethics Committee (21,787.001). All participants gave written informed consent.

### Participant characteristics and anthropometrics

Participant age, sex, ethnicity, Fitzpatrick skin type and Physical Activity Readiness-Questionnaire (PAR-Q) were recorded. Height was measured using a stadiometer (Seca217, Seca, Germany) to the closest centimetre. Weight was measured in kilograms using digital scales (Salter, UK).

### Study protocol

**Fig. 1:**
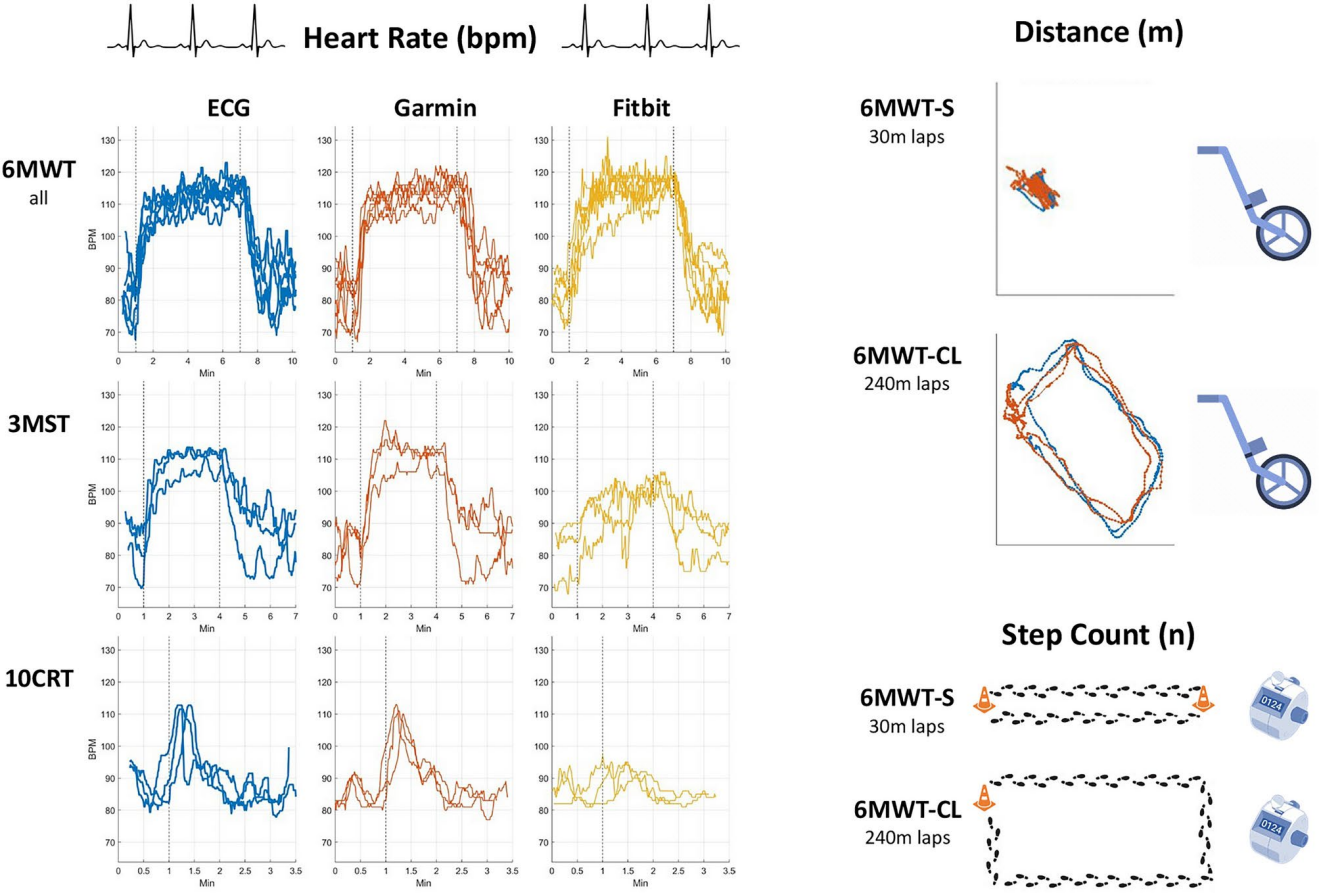
A schematic illustration of the study protocol including representative heart rate traces and GPS distance paths from one participant. 6MWT (6-min walk test), 6MWT-S (6MWT-standard), 6MWT-CL (6MWT- continuous lap), 3MST (3-min step test) and 10CRT (10 chair rise test).

Each participant was fitted with a Garmin Vivoactive4 and Fitbit Sense wrist-worn wearable to measure the following parameters: distance, step count and HR response. Allocation of each device to left or right wrist was randomized. Reference measures used to assess smartwatch accuracy were a meter-wheel for distance, hand tally counter (rounded to the closest 10 steps) for the number of steps, and ECG (Faros 180, Bittium) for heart rate. Each of the four tests (i.e. 6MWT-standard, 6MWT-continuous lap, 3MST and 10CRT) was repeated three times (a total of 12 tests per participant) across two clinic visits with an interval of at least 24 h between visits. The study protocol and typical results are shown in Fig. 1.

### Clinical tests of exercise capacity

6MWTs were conducted outdoors in Tavistock Square Gardens, London, after connecting the devices to GPS and starting a ‘walk’ smartwatch activity recording. Participants were instructed to cover as much distance as possible without running using two protocols: (1) a standard test (6MWT-standard) using straight 30 m laps. This mimics a clinical test which is usually conducted along a corridor. During this test study participants were directed to walk up and down a 30-m flat stretch marked by cones and (2) a non-standard test (6MWT-continuous lap), in which study participants were directed to walk freely around a park (240-m laps). This mimics a walk conducted in the community. On termination of the exercise phase, participants were asked to stand for a 3-min recovery phase.

3MSTs and 10CRTs were conducted indoors after starting a ‘cardio’ smartwatch activity recording which does not use the GPS. For the 3MST, following a 1-min standing resting phase, participants were invited to step up and down onto a single 20 cm step at a rate of 24 steps per minute using a metronome for 3-min. On termination of the exercise phase, participants were asked to stand for a 3-min recovery phase.

For the 10CRT, participants were instructed to sit centred on a chair with hands placed on the opposite shoulder with arms crossed. After a 1-min seated resting phase, participants were then asked to stand to full upright position and then sit back down again as quickly as possible for a total of 10 times. On termination of the exercise phase, participants were asked to sit for a 3-min recovery phase.

### Data analysis

Smartwatch data was exported from the Garmin Connect and Fitbit online portals using their respective application programming interface. ECG recordings were sampled at 1000 Hz and Garmin and Fitbit devices provided HR data every 1-s. Heart rate data was measured by ECG using bespoke software^18^. Mean HR was measured during three intervals in each test: (1) at rest (30 s prior to the onset of exercise); (2) at peak exercise (during the last minute of exercise in 6MWTs, and 3MSTs and from 60 to 105 s after the onset of 10CRTs) and (3) during recovery (from 30 s post exercise and for 1 min for 6WWT and 3MST and 45 s for 10CRT).

### Statistical analysis

Statistical analyses were performed using MATLAB 2022a, STATA 17.0 and R. Sample characteristics and reference measures are described using median [interquartile range, IQR] for continuous variables and frequency (percentage) for categorical variables. Concordance was assessed using Lin’s concordance correlation coefficient (CCC). Agreement was assessed using Bland–Altman analysis^19^ and results are presented as mean differences (limits of agreement [LoA]) accounting for repeated measures. Differences from reference values and absolute percentage error (APE) are reported as median [IQR]. Outliers were defined as measurements for which APE > 20%. Missing data were dealt with via listwise deletion, which is valid under the assumption of missing completely at random. Comparisons between device accuracy were conducted using two-way ANOVA tests. For HR, the effect of using Fitbit vs Garmin on APE was assessed pooling all data together and considering participants, test types (6MTW, 3MST and 10CRT), and test phases (rest, peak exercise, and recovery) as nested random effects. Comparisons for each test type and phase were also conducted using 2-way ANOVA, considering participants as random effects. APE of HR and steps were log-transformed to reduce right skewness. The association between absolute errors and participant characteristics was assessed using linear mixed-effect models to identify possible sources of inaccuracies. For HR, the models used the absolute errors in HR as the independent variable; participant sex, height, weight, and skin tone (Fitzpatrick scale) as fixed effects; participants IDs, test type (6MWT, 3MST and 10CRT) and test phases (rest, peak exercise, and recovery) as random effects. For steps and distance, which are only measured during 6MWT, random effects only included participant IDs. Continuous independent variables were normalized before entering the model.

## Results

### Participant characteristics

The characteristics of the 16 study participants are summarized in Table 1. Of the 16 adults recruited, 15 adults completed both study visits resulting in the following number of observations: 6MWT-standard n = 45, 6MWT-continuous lap n = 45, 3MST n = 45 and 10CRT n = 48. All 10CRT were completed in full, that is that a full chair rise was performed 10 times in total in serial in each test. Similarly, in all 3MST, 24 steps (up and down) were completed for a total of 3-min (72 steps in total). A summary of the number of participants, observations for each test and median [IQR] for the reference measure of each of the parameters assessed is provided in Table 2.

**Table 1:** Study participant characteristics. Data expressed as median [interquartile range, IQR] or frequency (%). BMI (body mass index).

Median [IQR] or n (%)	n = 16
Sex (male)	7 (44%)
Age (years)	27 [26, 29]
Height (cm)	170.5 [161.1,174.8]
Weight (kg)	67.3 [63.4,77.0]
BMI (kg/m^2^)	23.9 [22.4,25.7]
Fitzpatrick scale	2 [2,4]

**Table 2:** A summary of the number of participants, observations, and reference results for pooled 6-min walk tests (6MWT all), 6-min walk test—standard (6MWT-standard), 6-min walk test continuous lap (6MWT-continuous lap), 3-min step tests (3MST) and 10 chair rise tests (10CRT).

Test	Parameter	n	Obs	Median [IQR]
6MWT-standard	Distance	16	45	649 [604, 694]
	Step Count	16	45	800 [760, 840]
6MWT-continuous lap	Distance	16	45	679 [638, 746]
	Step Count	16	45	800 [760, 840]
6MWT all	Rest. HR	16	90	88 [80,102]
	Ex. HR	16	90	128 [115, 152]
	Rec. HR	16	90	103 [94, 125]
3MST	Rest. HR	16	45	81 [74, 96]
	Ex. HR	16	45	114 [102, 123]
	Rec. HR	16	45	96 [85, 110]
10CRT	Rest. HR	16	48	76 [65, 85]
	Ex. HR	16	48	99 [88, 107]
	Rec. HR	16	48	78 [70, 84]

Heart rate (HR) is measured in beats per minute, and distance is measured in metres. Results are presented as n or median [interquartile range]. resting (Rest.), peak exercise (Ex.) and 1-min recovery (Rec.).

### 6-min walk tests

#### Distance

**Fig. 2:**
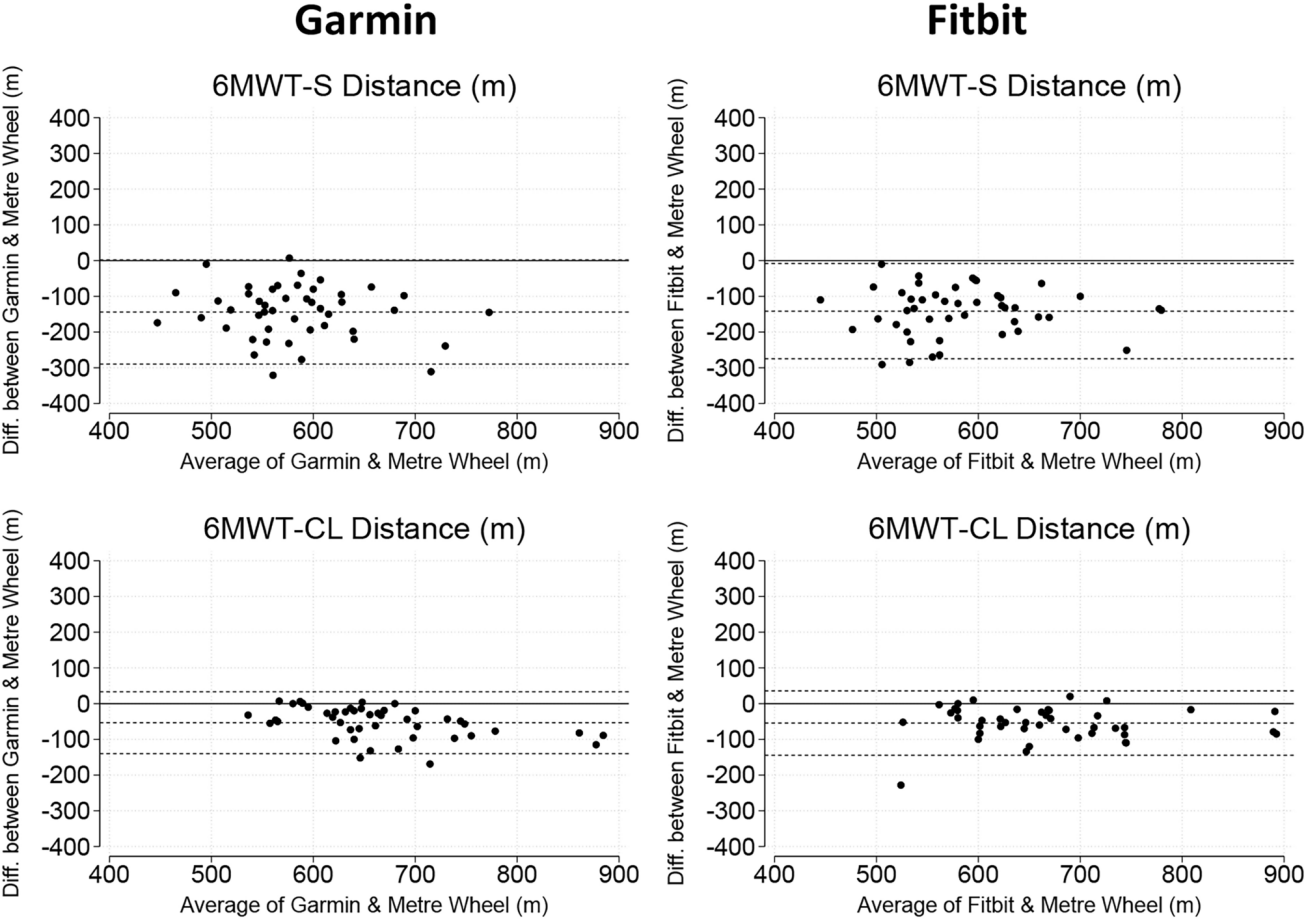
Bland–Altman plots demonstrating levels of agreement between smartwatch GPS distance and meter-wheel distance during 6-min walk test standard (6MWT-S) and 6-min walk test continuous lap (6MWT-CL).

Participants walked further during the 6MWT-continuous lap compared to the 6MWT-standard (6MWT-standard: 649 m [604, 694 m]; 6MWT-continuous lap: 679 m [638, 746 m], *p* < 0.001) (Table 2). Distance covered during the 6MWT-continuous lap was measured more accurately than during 6MWT-standard for both Garmin (6MWT-continuous lap: MAPE = 6.4% [3.0, 10.4%]; 6MWT-standard: MAPE = 20.1% [13.9, 28.4%], *p* < 0.001) and Fitbit (6MWT-continuous lap: MAPE = 8.0% [2.9, 10.1%]; 6MWT-standard: MAPE = 18.8% [15.2, 28.1%], *p* < 0.001), indicating that the 6MWT-continuous lap protocol is more suitable for remote monitoring (Table 3). MAPE for distance was not different between Garmin and Fitbit in either 6MWT-standard (20.1% [13.9, 28.4%] versus 18.8% [15.2, 28.1%], *p* = 0.935) or 6MWT-continuous lap (6.4% [3.0, 10.4%] versus 8.0% [2.9, 10.1%], *p* = 0.678) protocol, respectively. Bland–Altman plots showed that both Garmin and Fitbit smartwatches underestimated the distance walked in the walk tests, although this bias was greater for the 6MWT-standard than 6MWT-continuous lap (Fig. 2).

**Table 3:** Distance covered (metres) and step count (n) from the Garmin and Fitbit smartwatches during standard and non-standard 6-min walk tests (6MWT-standard & 6MWT-continuous lap) are compared to the reference measure values.

	Parameter	CCC	Diff. [LoA]	APE	Outliers
Garmin					
6MWT- standard	Distance	0.18	− 144 [− 290, 2]	20.1 [13.9, 28.4]	23 (51.1)
	Step Count	0.91	− 12 [− 66, 42]	1.8 [0.9, 2.9]	0 (0.0)
6MWT- continuous lap	Distance	0.71	− 54 [− 140, 33]	6.4 [3.0, 10.4]	2 (4.4)
	Step Count	0.88	− 11 [− 93, 71]	0.9 [0.4, 2.2]	1 (2.4)
Fitbit					
6MWT- standard	Distance	0.25	− 77 [− 205, 52]	18.8 [15.2, 28.1]	21 (46.7)
	Step Count	0.40	− 142 [− 275, − 8]	6.8 [3.2, 12.9]	4 (8.9)
6MWT- continuous lap	Distance	0.74	− 55 [− 145, 36]	8.0 [2.9, 10.1]	1 (2.3)
	Step Count	0.46	− 75 [− 216, 66]	8.0 [2.6, 12.3]	6 (13.6)

Lin’s concordance correlation coefficient (CCC), mean difference [limits of agreement], absolute percentage error (APE) and number of outliers (%) are presented.

### Step count

**Fig. 3:**
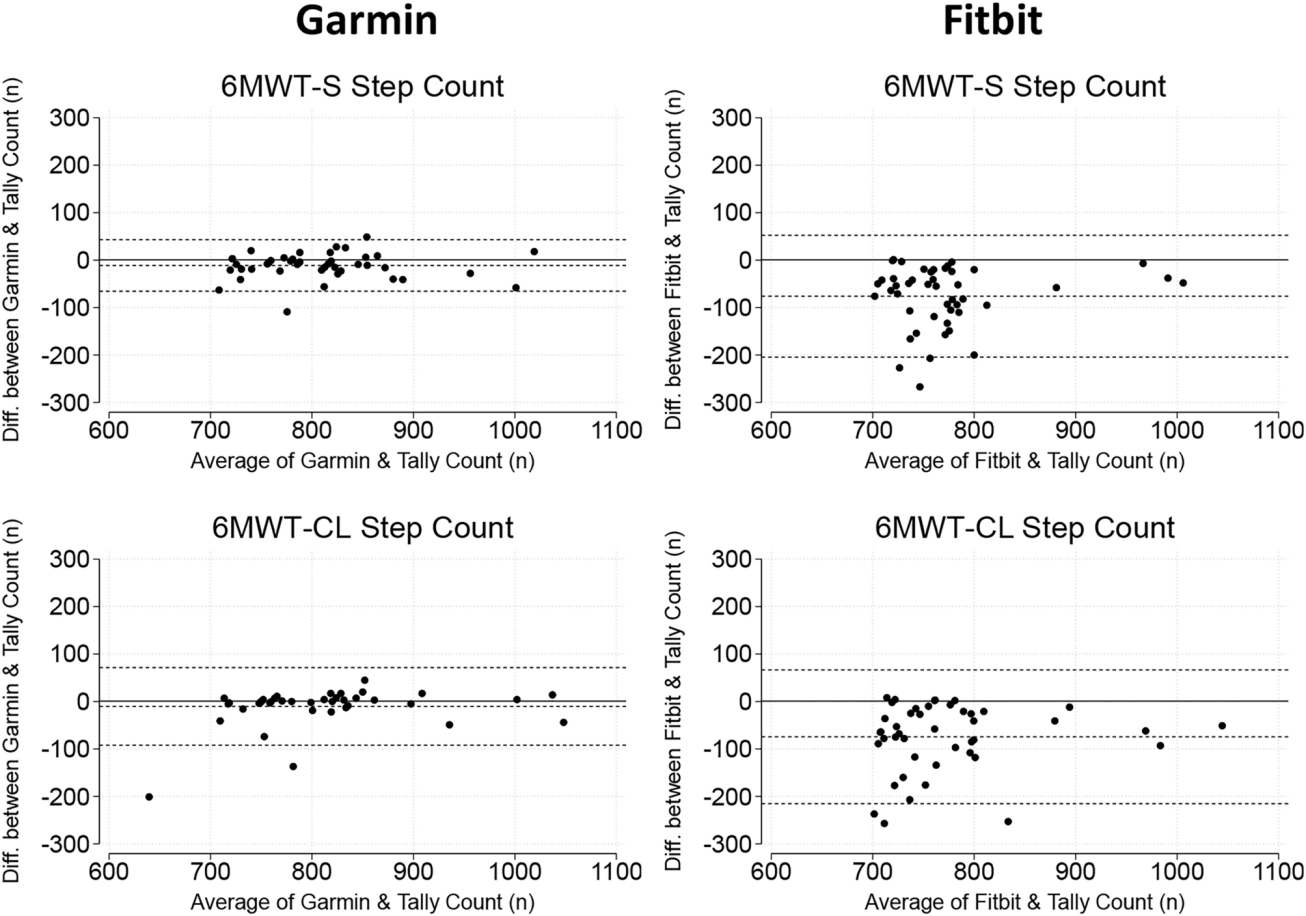
Bland–Altman plots demonstrating levels of agreement between smartwatch step count and hand tally count during 6-min walk test standard (6MWT-standard) and 6-min walk test continuous lap (6MWT-continuous lap).

Participants did a similar number of steps during the 6MWT-continuous lap compared to the 6MWT-standard (6MWT-standard: 800 steps [760, 840 steps]; 6MWT-continuous lap: 800 steps [760, 840 steps], *p* = 0.83) (Table 2). Differences were not observed in errors for step count between the two protocols (*p* > 0.9). Compared to the Fitbit, the Garmin device showed smaller errors for step count for both 6MWT-standard (Garmin: MAPE = 1.8% [0.9, 2.9%] and Fitbit: MAPE = 6.8% [3.2, 12.9%], *p* < 0.001) and 6MWT-continuous lap (Garmin: MAPE = 0.9% [0.4, 2.2%] and Fitbit: MAPE = 8.0% [2.6, 12.3%], *p* < 0.001) despite the same number of steps being performed (Tables 2 and 3). Bland–Altman plots for smartwatch step count and hand tally counter are shown in Fig. 3 and illustrate the better agreement for Garmin.

### Heart rate

**Fig. 4:**
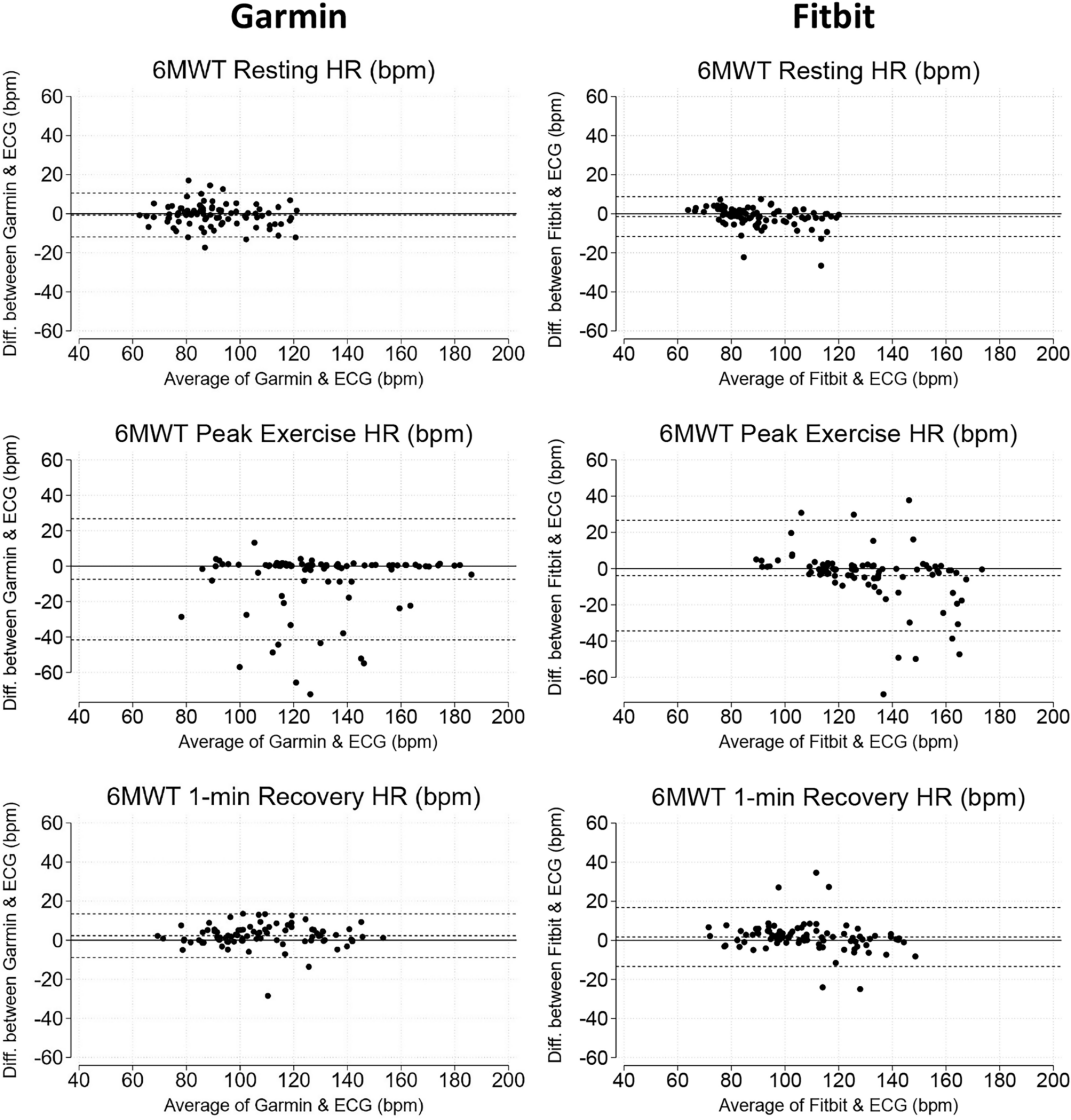
Bland–Altman plots demonstrating levels of agreement between smartwatch heart rate (HR) and ECG HR during pooled 6-min walk tests (6MWT).

HR results are presented as a pooled analysis for 6MWT protocols (Tables 2 and 4). Both devices showed small median errors when measuring HR at rest (Garmin: MAPE = 3.1% [1.1, 6.1%] and Fitbit: MAPE = 2.3% [1.4, 5.3%], *p* = 0.180), peak exercise (Garmin: MAPE = 0.8% [0.3, 6.0%] and Fitbit: MAPE = 2.5% [1.1, 7.3%], *p* = 0.003 and recovery (Garmin: MAPE = 3.1% [1.1, 5.6%] and Fitbit: MAPE = 3.2% [1.3, 5.3%], *p* = 0.503). Bland–Altman plots are shown in Fig. 4. These showed little evidence of bias under any condition, but the LOA increased noticeably during peak exercise.

**Table 4:** Lin’s concordance correlation coefficient (CCC), Bland–Altman plots for repeated measures presented as mean difference [limits of agreement], absolute percentage error (APE) presented as median [interquartile range] and number of outliers (%) results for pooled 6-min walk tests (6MWT), 3-min step tests (3MST) and 10 chair rises tests (10CRT) heart rate (HR) at rest (Rest.), peak exercise (Ex.) and 1-min recovery (Rec.).

	Parameter	CCC	Diff. [LoA]	APE	Outliers
Garmin					
6MWT all	Rest. HR	0.92	− 1 [− 12, 11]	3.1 [1.1, 6.1]	1 (1.1)
	Ex. HR	0.74	− 8 [− 42, 27]	0.8 [0.3, 6.0]	12 (13.3)
	Rec. HR	0.95	2 [− 9, 14]	3.1 [1.1, 5.6]	1 (1.1)
3MST	Rest. HR	0.93	− 2 [− 12, 9]	2.4 [1.1, 5.7]	1 (2.2)
	Ex. HR	0.38	11 [− 24, 46]	0.5 [0.2, 22.5]	13 (28.9)
	Rec. HR	0.79	6 [− 15, 26]	2.6 [1.1, 9.6]	6 (13.3)
10CRT	Rest. HR	0.96	− 1 [− 7, 5]	1.9 [1.0, 3.9]	0 (0.0)
	Ex. HR	0.75	− 7 [− 20, 5]	7.1 [1.4, 12.6]	3 (6.2)
	Rec. HR	0.95	1 [− 6, 9]	1.4 [0.5, 3.8]	0 (0.0)
Fitbit					
6MWT all	Rest. HR	0.93	− 2 [− 12, 9]	2.3 [1.4, 5.3]	2 (2.2)
	Ex. HR	0.75	− 4 [− 34, 27]	2.5 [1.1, 7.3]	9 (10.0)
	Rec. HR	0.91	2 [− 13, 17]	3.2 [1.3, 5.3]	3 (3.3)
3MST	Rest. HR	0.96	0 [− 8, 9]	3.0 [1.2, 5.0]	1 (2.2)
	Ex. HR	0.61	− 10 [− 39, 18]	10.3 [5.2, 15.5]	7 (15.6)
	Rec. HR	0.94	0 [− 11, 12]	3.2 [1.7, 6.2]	0 (0.0)
10CRT	Rest. HR	0.79	1 [− 12, 13]	2.1 [1.0, 4.0]	2 (4.2)
	Ex. HR	0.43	− 13 [− 26, 0]	12.1 [9.1, 17.4]	5 (10.4)
	Rec. HR	0.96	0 [− 6, 6]	2.2 [1.5, 3.6]	0 (0.0)

### 3-min step tests

**Fig. 5:**
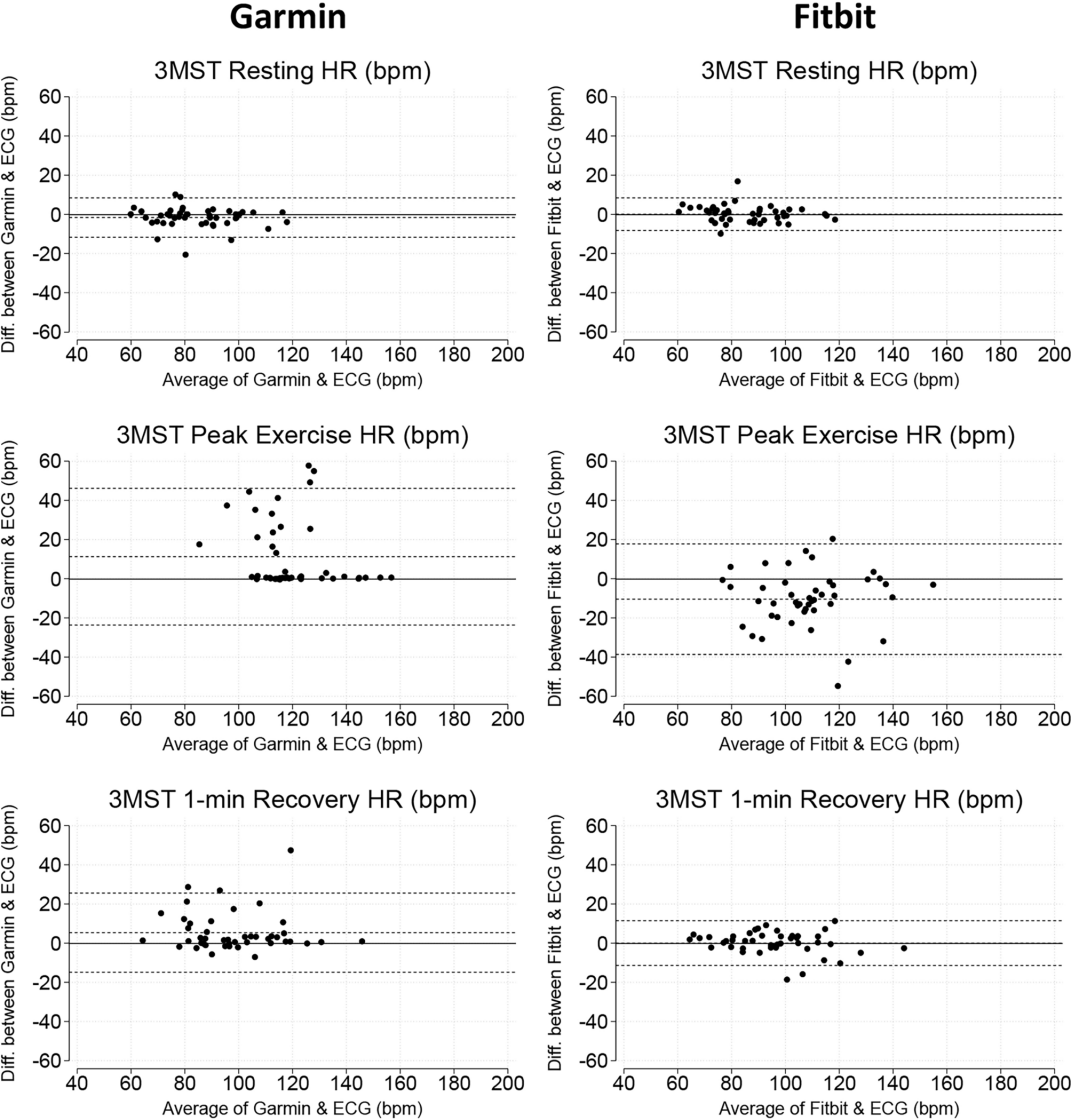
Bland–Altman plots demonstrating levels of agreement between smartwatch heart rate (HR) and ECG HR during 3-min step tests (3MST).

Both devices showed small errors in measuring HR at rest (Garmin: MAPE = 2.4% [1.1, 5.7%] and Fitbit: MAPE = 3.0% [1.2, 5.0%], *p* = 0.898) and recovery (Garmin: MAPE = 2.6% [1.1, 9.6%] and Fitbit: MAPE = 3.2% [1.7, 6.2%], *p* = 0.946). Error during peak exercise for Garmin was MAPE = 0.5% [0.2, 22.5%] and for Fitbit was MAPE = 10.3% [5.2, 15.5%], *p* < 0.001 (Table 4). The relevant Bland–Altman plots are shown in Fig. 5.

### 10-chair rise tests

**Fig. 6:**
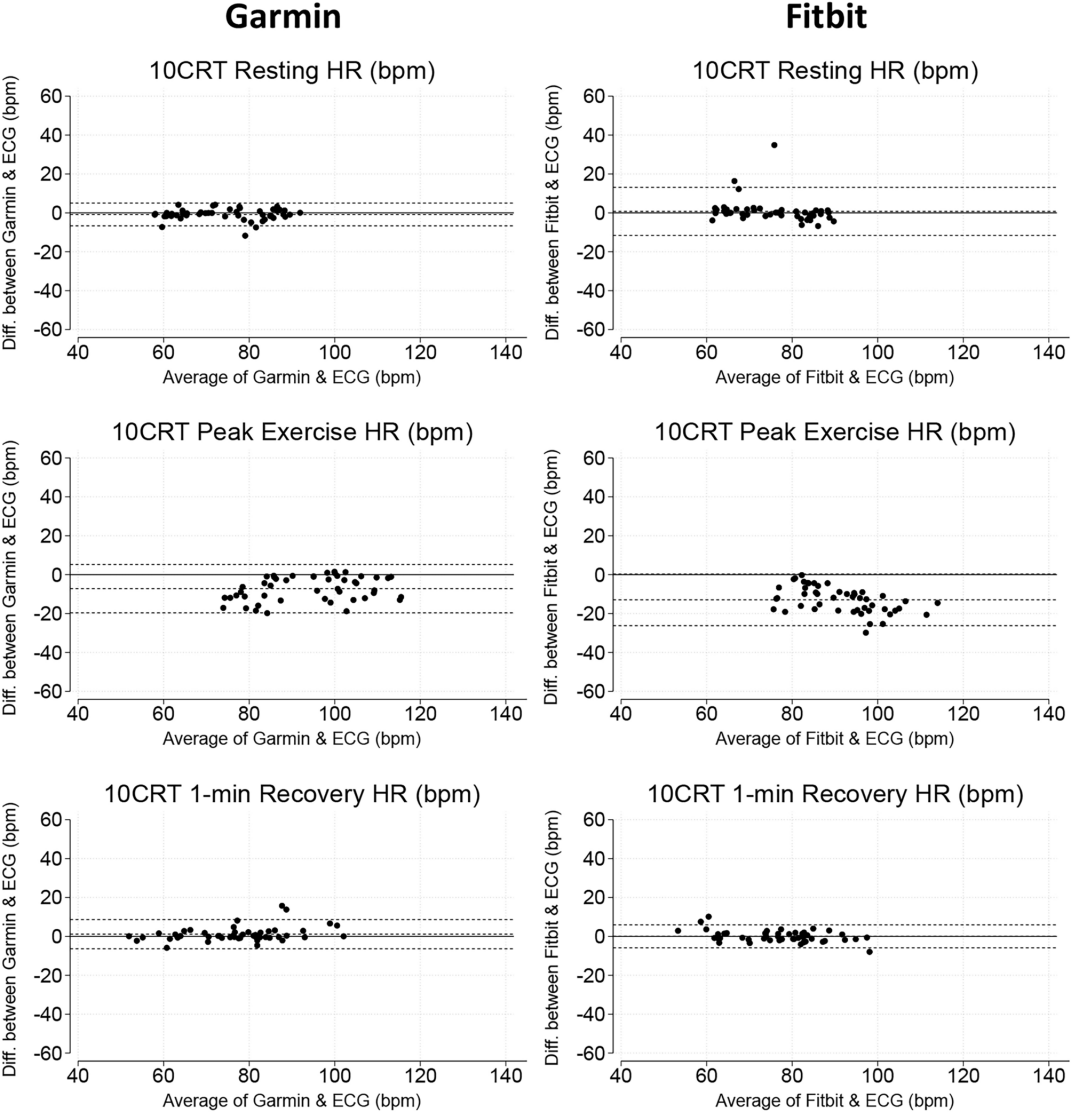
Bland–Altman plots demonstrating levels of agreement between smartwatch heart rate (HR) and ECG HR during 10 chair rises test (10CRT).

Both devices showed small errors in measuring HR at rest (Garmin: MAPE = 1.9% [1.0, 3.9%] and Fitbit: MAPE = 2.1% [1.0, 4.0%], *p* = 0.533) and recovery (Garmin: MAPE = 1.4% [0.5, 3.8%] and Fitbit: MAPE = 2.2% [1.5, 3.6%], *p* value = 0.115). Median error during peak exercise for Garmin was MAPE = 7.1% [1.4, 12.6%] and for Fitbit was MAPE = 12.1% [9.1, 17.4%], *p* < 0.001 (Table 4). Bland–Altman plots illustrating the limits of agreement between smartwatch HR and ECG HR are shown in Fig. 6.

Lin’s CCC, Bland–Altman analysis results, APE and number of outliers results are summarized for HR measured in all tests in Table 4.

### Comparison of HR inaccuracies across devices

After pooling together 1098 observations across two devices (Garmin and Fitbit), 15 participants, three test types (6MWT, 3MST, 10CRT), six repetitions for 6MWT, three repetitions for 3MST and 10CRT, three test phases (rest, peak exercise, 1-min recovery) and accounting for pseudo-replication using a two- way ANOVA model with nested random effects, Garmin was found have lower APE than Fitbit (*p* < 0.001), with differences driven by lower APE during peak exercise.

### Sources of inaccuracy

We found no convincing evidence of associations between absolute errors in HR or distance and participant characteristics (Table 5). A weak association between height and step count errors for the Fitbit device may have been a chance finding given the number of comparisons examined.

**Table 5:** Univariate associations between absolute errors and selected participant characteristics for heart rate, step count and distance.

	Garmin		Fitbit	
	Heart rate errors		Heart rate errors	
	β (95% CI)	*P*	β (95% CI)	*P*
Male sex	2.5 (− 0.4, 5.5)	0.095	− 0.20 (− 2.32, 1.92)	0.850
Height	0.6 (− 1.0, 2.2)	0.463	− 0.20 (− 1.25, 0.86)	0.716
Weight	− 0.5 (− 2.1, 1.1)	0.510	− 0.03 (− 1.09, 1.03)	0.957
Fitzpatrick	− 0.6 (− 2.2, 1.0)	0.473	− 0.60 (− 1.61, 0.42)	0.250
	Step count errors		Step count errors	
	β (95% CI)	P	β (95% CI)	P
Male sex	− 13.04 (− 25.97, − 0.12)	0.048	− 18.06 (− 71.17, 35.05)	0.501
Height	− 0.31 (− 1.13, 0.50)	0.446	− 2.48 (− 4.84, − 0.12)	0.039
Weight	0.19 (− 0.55, 0.93)	0.609	− 2.05 (− 4.20, 0.09)	0.061
Fitzpatrick	2.24 (− 4.72, 9.20)	0.523	− 1.86 (− 24.14, 20.41)	0.868
	Distance errors		Distance errors	
	β (95% CI)	P	β (95% CI)	P
Male sex	17.37 (− 14.24, 48.98)	0.278	25.29 (− 4.56, 55.13)	0.096
Height	9.48 (− 6.14, 25.10)	0.231	9.47 (− 5.56, 24.50)	0.214
Weight	− 1.78 (− 17.52, 13.97)	0.823	− 4.09 (− 19.07, 10.89)	0.589
Fitzpatrick	− 7.15 (− 22.69, 8.40)	0.363	− 11.80 (− 26.46, 2.85)	0.113

Results are presented as regression coefficients, β (95% confidence interval) and respective *p* values

## Discussion

The aim of this study was to establish the accuracy of two state-of-the-art consumer grade smartwatches for distance, step count and HR during three established sub-maximal clinical assessments of exercise capacity (6MWT, 3MST and 10CRT) in a young and healthy population. The standardized tests selected in this study are relatively easy to perform, do not require the provision of specialist equipment and could be performed without supervision, providing an opportunity to be able to monitor exercise capacity frequently and at scale.

The main results of the study were (1) Compared to the gold-standard meter-wheel reference, distance measured by both wrist-worn devices (Garmin & Fitbit) was accurate, with as little as 6–8% error, during a non-standard 6MWT protocol. However, error increased to 18–20% when a standard 30 m lap 6MWT protocol was used. (2) Step count was a more accurate measure of distance compared to GPS distance (MAPE: 0.9%[0.4, 2.2%] and 6.8% [3.2, 12.9%] for Garmin and Fitbit, respectively), and did not substantially differ using a standard or a continuous lap 6MWT protocol. (3) Both devices demonstrated excellent accuracy in measuring HR at rest, and during recovery (MAPE ≤ 3%), but accuracy worsened during peak exercise. MAPE was similar to rest and recovery during peak exercise, however, the limits of agreement widened due to an increase in the number of outliers (~ 7% for Garmin and ~ 12% for Fitbit).

Performing a remote 6MWT in the home environment may be limited due to the short track lengths available, therefore, we assessed walk tests outdoors in a park. 6MWT were performed in the traditional manner, i.e. back and forth along straight 30 m paths, and also using continuous circular laps which is more similar to ‘free-range’ walking. Both devices had smaller errors of distance for the 6MWT-continuous lap protocol (median MAPE ≤ 8%) compared to the 6MWT-standard protocol (median MAPE ≤ 20%). Distance is measured by these devices through the activation of GPS and the greater errors seen in the 6MWT-standard are likely due to the use of short 30 m stretches which requires time spent turning and a frequent number of turns per test. All tests were performed in an inner city which in turn may contribute to underestimation of GPS-measured distance, however, this reflects realistic scenarios of remote monitoring.

Step count is measured by both devices using a composite of stride length (estimated by pre-programmed height) and tri-axial accelerometery data. In agreement with Rens and colleagues’ findings, we observed better accuracy in step count compared to GPS distance^16^. This finding likely also relates to the turning requirement in 6MWT-standard and the small pivot steps required to do so which may be underestimated by the devices. The Garmin device performed better than the Fitbit with a median APE of < 2% compared with ~ 8% for Fitbit.

Wearables also offer the opportunity to measure chronotropic responses to these exercise tests. Resting HR, HR at peak exercise and HR recovery following exercise are associated with mortality^20–22^. Irrespective of test, both devices demonstrated good HR accuracy and agreement at rest and recovery, with MAPE ≤ 3%. The number of outliers, defined as recordings showing an absolute percentage error > 20%, was small (< 5%) during rest, but it increased to 10–30% during peak exercise. This indicates that while the majority of measurements taken during peak exercise are accurate, the frequency of substantially incorrect measures increases. This increase may be due to reduced contact pressure between the device sensor and the wrist, sweating and/or increase in motion artifacts due to arm movements during walking^23^. Arm movements may also explain why the number of outliers was larger during the 3MST than the 6MWT despite a lower HR increase. It is possible that smartwatches may be more robust to the type of arm movement typically observed during walking, than during a step test.

In line with our findings, prior work has demonstrated that smartwatches can measure pulse rate accurately at rest, with reductions in accuracy reported during physical activity such as walking, treadmill exercise and cycling^24–26^. Furthermore, step count validity has previously been shown to be more accurate than GPS measured distance^27^. One limitation of our study is that the battery of tests investigated are all sub-maximal assessments of exercise capacity and therefore do not measure maximal cardiorespiratory fitness, which should be assessed using cardiopulmonary exercise testing (CPET). A comparison of these performance findings and heart rate responses to gold-standard CPET measured outcomes would be an interesting future direction.

Creagh and colleagues^28^ utilized Motorola 360 Sport smartwatches and Samsung Galaxy S7 smartphones to assess whether signal-based features extracted from a remote 2-min Walk Test could distinguish individuals with multiple sclerosis from healthy controls^28^. They reported that the two groups could be discriminated with an accuracy of 82 ± 2%, (sensitivity of 80 ± 4% and specificity of 87 ± 3%). We are unaware of previous studies that have validated the use of smartwatches in commonly used and standardized submaximal tests used clinically or in large epidemiological studies.

### Limitations

One limitation of this study is its small sample size; however, participants were asked to repeat each test several times to obtain up to 90 comparisons per test. The age and healthy condition of participants may also be considered a limitation; the tests of exercise capacity investigated in this study are usually performed clinically in older populations with either cardiopulmonary or neuromuscular dysfunction, where slower movement may negatively impact the accuracy of distance tracking and step counting^29^. The step distances for the 6MWT-standard and 6MWT-continuous lap were 649 and 679, respectively. These distances are known to drop into the mid 500 range for a 60 + year old population, therefore, replication of these analyses in the noted clinical populations would be an important next step. Furthermore, the younger population used in this study may have handled the turning radius and overall agility required to turn in a standard 6MWT more efficiently than the population that would typically be invited to perform this test. Therefore, the error noted in the standard 6MWT may be enhanced in an aged population.

## Conclusions

In this study of young and healthy individuals, pulse rate dynamics during walk, steps and chair rise tests can be measured with acceptable accuracy using two consumer grade smartwatches. This method has the potential to be used to assess exercise capacity remotely in the community. The use of a continuous lap is preferred to the standard back and forth track when assessing the 6MWT using smartwatches. Generally, in this study, Garmin Vivoactive 4 provided more accurate measures than Fitbit Sense. Further studies are required to evaluate remote assessment of exercise capacity in individuals with cardiorespiratory conditions or frailty.

## Data Availability

The datasets generated and/or analysed during the current study are not publicly available due to the sensitive nature of the data collected but are available from the corresponding author on reasonable request.
